# Proteobacteria: A Toolbox for Clinicians

**DOI:** 10.3390/microorganisms14102238

**Published:** 2026-10-03

**Authors:** William Fusco, Ernesto Margarita, Enrico Capuano, Andrea Severino, Serena Porcari, Simone Varca, Tiziano Galasso, Elena Lener, Antonio Gasbarrini, Gianluca Ianiro

**Affiliations:** 1 Department of Translational Medicine and Surgery, Università Cattolica del Sacro Cuore, 00168 Rome, Italysevandrea96@gmail.com (A.S.); antonio.gasbarrini@policlinicogemelli.it (A.G.); 2 Department of Medical and Surgical Sciences, UOC CEMAD Centro Malattie dell’Apparato Digerente, Medicina Interna e Gastroenterologia, Fondazione Policlinico Universitario Gemelli IRCCS, 00168 Rome, Italy; 3 Department of Medical and Surgical Sciences, UOC Medicina Interna e Gastroenterologia, Fondazione Policlinico Universitario Gemelli IRCCS, 00168 Rome, Italy

**Keywords:** gut microbiota, proteobacteria, microbiome modulation

## Abstract

Proteobacteria are a phylum of Gram-negative bacteria that, although normally a minor component of the healthy gut microbiota, can be markedly increased in many conditions. Their overgrowth is in fact linked to inflammation, impaired epithelial barrier function, and diseases such as inflammatory bowel disease, metabolic syndrome, and gastrointestinal cancers. Key species, like *Escherichia coli* and *Helicobacter pylori*, can promote disease through specific etiopathogenetic mechanisms, from genotoxin production to immune activation: this peculiarity can be used for diagnostic purposes, and in some cases even as therapeutics. Emerging microbiota-targeted therapies—such as dietary modulation, probiotics, fecal microbiota transplantation, bacteriophages, and engineered bacteria—aim to restore microbial balance. This narrative review deals with the dual role of Proteobacteria as both pathogens and diagnostic–therapeutic tools, which is crucial for advancing personalized microbiome-based medicine.

## 1. Introduction

Proteobacteria (although currently and more accurately known as Pseudomonadota), a metabolically versatile and phylogenetically diverse phylum of Gram-negative bacteria, play both beneficial and pathogenic roles in human health [1]. Comprising eight major classes [2], they typically include a minor fraction of the healthy human gut microbiota, as well as different pathogenic, conditionally pathogenic, or opportunistic species. However, their overrepresentation is often a marker of dysbiosis and is associated with conditions such as inflammatory bowel diseases (IBDs), metabolic syndrome, and cancer [3,4,5].

Their success in hostile or inflamed environments is due to metabolic adaptability and genomic plasticity [6].

Notably, their evolutionary significance is underscored by the origin of mitochondria from an ancestral Alphaproteobacterium [7], reflecting their deep connection to eukaryotic life.

Despite these beneficial aspects, many Proteobacteria are opportunistic or pathogenic. Species like *Escherichia coli*, *Helicobacter pylori*, and *Klebsiella pneumoniae* are linked to infections and chronic non-communicable diseases (NCDs). In a balanced gut ecosystem, obligate anaerobes such as many of the Firmicutes (currently known as “Bacillota”) and Bacteroidetes (currently known as “Bacteroidota”) predominate, while Proteobacteria remain scarce. Inflammation disrupts this balance, increases oxygen levels, and supports the growth of facultative anaerobes like Enterobacteriaceae, a shift known as the “oxygen hypothesis” [8], which further aggravates inflammation.

Certain strains, including adherent-invasive *E. coli* (AIEC), contribute to IBD pathogenesis through epithelial adhesion, invasion, and immune activation [3]. Genotoxin-producing *E. coli* strains with colibactin can promote colorectal cancer (CRC) by inducing DNA damage and pro-inflammatory pathways [4].

Proteobacteria are also involved in metabolic disorders such as metabolic dysfunction-associated steatotic liver disease (MASLD, formerly non-alcoholic fatty liver disease, NAFLD) and metabolic syndrome [5]. Their overgrowth is linked to systemic inflammation and metabolic dysfunction. Lipopolysaccharides (LPSs), key components of their outer membrane, activate TLR4-mediated immune responses, bridging gut dysbiosis with liver inflammation and insulin resistance [9]. Moreover, some strains produce endogenous alcohol, which can exacerbate liver injury, as seen in autobrewery syndrome (ABS) [10].

Their impact extends to gastric cancer, where *H. pylori* remains the major risk factor due to virulence factors like CagA and VacA that drive inflammation, immune evasion, and oncogenesis [11]. Other Proteobacteria, including Enterobacteriaceae, may also contribute by generating reactive oxygen species and carcinogenic metabolites in the gastric environment [12].

In recent decades, emerging microbiota-targeted therapies—such as tailored probiotics, dietary interventions, bacteriophage therapy, and engineered bacteria—have increasingly been developed to counteract Proteobacteria overgrowth, restore microbial equilibrium, and mitigate inflammation [13].

This narrative review aims to be a guide for clinicians considering the medical relevance of the Proteobacteria phylum. Specifically, it will explore the taxonomy, physiology, and pathogenicity of Proteobacteria, emphasizing their involvement in inflammation, metabolic diseases, and cancer, and examining therapeutic strategies to restore microbial balance, highlighting the dual nature of Proteobacteria as both a target and a tool in modern medicine, giving the clinician a tool for microbiome-based clinical practice.

## 2. Taxonomic Profile and Physiological Characteristics of Proteobacteria

Proteobacteria, a phylum of Gram-negative bacteria [14], have been recognized as essential to early gut colonization. They typically represent only a minor fraction of the gut microbiota under healthy conditions [15]; their expansion is a hallmark of dysbiosis and is linked to inflammatory, infectious, and metabolic diseases [16]. According to the most recent classification, Proteobacteria are divided into eight monophyletic classes: Alphaproteobacteria, Betaproteobacteria, Gammaproteobacteria, Deltaproteobacteria, Epsilonproteobacteria, Acidithiobacillia, Hydrogenophilia, and Oligoflexia (while previously a “Zetaproteobacteria” class was also present) [2,17] (Figure 1). Among them, Alphaproteobacteria include genera such as *Rickettsia*, *Brucella*, *Bartonella*, *Ehrlichia,* and *Anaplasma*, many of which are intracellular pathogens and oligotrophs capable of surviving in nutrient-poor environments [18,19,20]. Betaproteobacteria contain medically relevant genera such as *Bordetella* and *Neisseria*, responsible for pertussis, gonorrhoea, and meningitis [21,22]. Gammaproteobacteria comprise diverse genera such as *Escherichia*, *Pseudomonas*, *Haemophilus*, *Vibrio*, *Klebsiella*, *Shigella*, and *Salmonella*, many of which are human pathogens [23,24]. In contrast, Deltaproteobacteria is a smaller class, with *Desulfomicrobium orale* associated with periodontal disease [25], while Epsilonproteobacteria—the smallest class—includes *Campylobacter* and *Helicobacter*, both of clinical importance [26].

Proteobacteria also play a vital role in eukaryotic evolution. The widely accepted endosymbiontic origin of mitochondria from an ancestral Alphaproteobacterium is supported by genetic similarities with mitochondrial and cytosolic proteins [7,27,28,29,30,31].

In neonates, Proteobacteria are early colonizers of the gut, consuming oxygen and promoting the establishment of obligate anaerobes [32,33,34]. Factors such as maternal diet, antibiotics, and mode of delivery influence microbiota composition [35,36], while their presence in the placenta and meconium suggests possible in utero transmission [37,38,39,40]. After birth, feeding mode and gestational age likely modulate Proteobacteria persistence: different studies suggest that breastfed infants might have more *Bifidobacterium*, while formula-fed ones seem to show higher levels of Enterobacteriaceae, likely due to the immunomodulatory effects of human milk oligosaccharides and secretory IgA [41,42,43,44,45].

Preterm infants often harbor greater Proteobacteria abundance [46], a key feature linked to necrotizing enterocolitis (NEC), a severe inflammatory condition marked by reduced microbial diversity and overgrowth of Enterobacteriaceae [47,48,49]. Normally, Proteobacteria decline after initial colonization; deviations from this pattern may increase the risk of neonatal diseases [48,49].

In adults, Proteobacteria dysbiosis is associated with various chronic conditions [50]. Their overrepresentation, especially of Enterobacteriaceae, is common in IBDs, notably Crohn’s disease (CD) [51,52], and the World Health Organization (WHO) classifies them as major threats due to rising antibiotic resistance [53,54]. *H. pylori*, a member of this phylum, is a well-established cause of peptic ulcers, gastric cancer, and mucosa-associated lymphoid tissue (MALT) lymphoma [55,56,57,58]. Additionally, genera like *Sutterella* are enriched in obesity and metabolic syndrome [59,60], and have been consistently detected in the gut microbiota of individuals with autism spectrum disorder [61].

Table 1 provides a schematic overview of the established Proteobacteria species associated with gastrointestinal and systemic disease [25,62,63,64,65,66,67,68,69,70,71,72,73,74,75,76,77,78,79,80,81,82,83,84,85,86,87,88,89,90,91]. The pathogenetic mechanisms of these partly microbiome-driven diseases and the role played by the single taxa are discussed in the following paragraphs.

## 3. Proteobacteria and Their Role in Inflammation and Carcinogenesis

An increasing body of literature provides evidence concerning the role of Proteobacteria as key drivers of gut and systemic inflammation, thereby contributing to the onset of inflammatory disorders, including IBDs and metabolic syndrome [51,92].

Different preclinical studies imply that increased Proteobacteria abundance is associated with an inflammatory environment [93]. By evaluating mice lacking the Toll-like receptor 5 (TLR5) (T5KO)—which are constitutively prone to inflammation—Carvalho et al. have indeed revealed augmented levels of this phylum, coupled with enhanced expression of proinflammatory interleukins (ILs) [93].

Consistent with this finding, patients with evidence of intestinal inflammation show an overgrowth of Proteobacteria [92,94,95], which may, in turn, impair intestinal permeability [96]. This might ultimately lead to bacterial translocation across the mucosal barrier, thus eliciting inflammation at a systemic level [96]. Taken together, these studies imply the entanglement between inflammation and proliferation of Proteobacteria—although mechanistic relations remain unclear to date. With respect to this, the so-called “oxygen hypothesis” suggests that oxygen consumption decreases when inflammation is triggered, due to an epithelial metabolic shift from beta-oxidation of butyrate to anaerobic glycolysis; the resulting increase in gut oxygenation determines a fall in obligate anaerobes, with concurrent blooming by facultative anaerobes, including Proteobacteria [97,98].

In this dysbiotic context, Proteobacteria may enhance inflammatory activity, through LPS expression [99] and gut epithelial invasion, which promotes secretion of pro-inflammatory cytokines, such as TNF-α and IFN-γ [100]. Moreover, it has recently been suggested that, along with a depletion in Ruminococcaceae, an expansion of bacteria belonging to the Enterobacteriaceae family may disrupt secondary bile acid metabolism [101], thus worsening inflammation. By engaging Takeda G protein-coupled receptor 5 (TGR5), these cholesterol derivatives indeed show immunoregulatory properties, by inhibiting the production of proinflammatory cytokines [102]. Overall, these findings hint that this phylum is deeply linked with inflammation in a bidirectional interplay.

Furthermore, mounting evidence puts emphasis on Proteobacteria as important players in human carcinogenesis, through both inflammation and direct cell damage [103,104]. As for the latter, different bacterial species have become able to produce a broad group of genotoxins, thus gaining a crucial evolutionary and ecological advantage against other microbes [105]. Similarly, specific members of the Enterobacteriaceae family, including *E. coli* and *K. pneumoniae*, secrete diverse peptides that disrupt the eukaryotic cell cycle, including cyclomodulins [106]; although their physiological role remains unclear, cyclomodulins are thought to modulate immunity, by shaping intestinal permeability during neonatal life [107]. As a consequence, an enrichment in Proteobacteria may lead to an overexpression of toxins—including colibactin and cytolethal distending toxin (CDT)—thereby inducing double-strand breaks in colonocytes’ genome. This may ultimately lead to genomic instability and increased risk of developing cancer [107].

Moreover, Meza-Perez et al. demonstrated that GF mice show stronger tumor rejection potential, compared to the conventional ones. The authors specifically observed that arginine levels were increased in the first group, due to the lower abundance of Proteobacteria [108], whose role in the consumption of this amino acid is well established [109]. Arginine, in turn, was revealed to impair regulatory T cells’ (Tregs) suppressive properties by eliciting the mTor pathway [108]. Consistent with these observations, supplementation of *E. coli* was demonstrated to restore proper Treg activity, an effect which could not be reproduced when administering a distinct strain unable to catabolize arginine. As a result, enrichment in Proteobacteria may determine a drop in arginine levels, thereby enhancing Tregs’ activity and disrupting cancer immunosurveillance.

Though essentially preliminary, taken together these findings underscore the need for further mechanistic studies in order to better explore not just the relationship, but specifically the causes of the relationship between Proteobacteria, inflammation, and carcinogenesis.

## 4. Proteobacteria-Associated Diseases

Understanding the role of Proteobacteria in human diseases requires exploring their contribution not only to chronic inflammatory conditions, but also to metabolic and oncological ones.

An increased abundance of Proteobacteria, especially members of the Enterobacteriaceae family, has been repeatedly observed in individuals with IBDs [110], with dysbiosis correlating with both disease activity and progression [111,112].

*E. coli* is one of the major species whose levels are well known to be enriched in patients with CD and UC [113]. In this regard, cumulating clinical evidence is highlighting adherent-invasive *E. coli* (AIEC) as a key pathogenic strain, capable of adhering to and invading epithelial cells in patients with CD through the interaction between type 1 pili and CEACAM6 [114]. Once intestinal mucosa has been colonized, AIEC seems to enhance TNF-α production by macrophages, thus exacerbating inflammation at both local and systemic levels [115]. Likewise, other *E. coli* strains, including extraintestinal pathogenic *E. coli* (ExPEC), seemingly disrupt the intestinal barrier through specific virulence factors—such as α-hemolysin—ultimately leading to an increase in intestinal permeability and consequent translocation of bacterial antigens across the mucosa [116,117]. This systemic antigenic exposure may, in turn, lead to IBDs’ well-known extraintestinal manifestations [118].

Finally, recent evidence suggests that some Proteobacteria known to also be present in the oral cavity—mainly *Klebsiella* spp.—may contribute to gut inflammation in patients with CD, by fostering Th1 activity [119,120]. Ectopic colonization by these bacteria via the oral–gut axis might be causally associated with periodontitis, which is consistently common in this setting [121].

In colorectal cancer (CRC), different clinical studies exploiting next-generation sequencing have documented a dysbiotic condition marked by increased Proteobacteria and reduced Firmicutes, particularly short-chain fatty acids (SCFAs) producing bacteria [122,123]. Within this framework, species such as genotoxin-producing *E. coli* can contribute directly to carcinogenesis. This has fueled the interest towards the role of Proteobacteria in colorectal carcinogenesis, leading to the formulation of two major hypotheses. The “driver-passenger” model proposes a two-stage mechanism: genotoxic bacteria (e.g., colibactin-producing *E. coli*) initiate DNA damage and inflammation, setting the stage for secondary colonization by other tumor-promoting microbes [123]. Alternatively, the “concerted effort” model suggests that *E. coli*-induced carcinogenesis may occur following initial microenvironmental changes by species like *Bacteroides fragilis* [124]. Thus, the potential Proteobacteria-mediated colorectal carcinogenesis appears to result from a combination of inflammation, microbiota remodeling, and direct DNA damage.

There is longstanding knowledge regarding *H. pylori* as an etiological agent of gastric cancer [125]. Besides its virulence factors’ (VacA, CagA, and peptidoglycan) direct role in oxidative stress and DNA damage [125], CagA has been shown in vitro to disrupt the Wnt/β-catenin pathway, which contributes to cell cycle dysregulation and gastric tumorigenesis [126]. Moreover, chronic *H. pylori* infection suppresses gastric acid secretion, promoting Proteobacteria overgrowth and reducing Firmicutes and Bacteroidetes [127,128,129]. This altered environment ultimately promotes the production of ROS and N-nitrosamines, thereby increasing the risk of developing gastric adenocarcinoma [130,131] (Figure 2). Apart from *H. pylori*, different clinical studies show that gastric cancer bioptical samples contain a higher abundance of Proteobacteria, including *Phyllobacterium* spp., *Achromobacter* spp., and members of Xanthomonadaceae and Enterobacteriaceae [132]. These taxa may serve as future biomarkers for disease diagnosis and prognosis.

An increase in Proteobacteria abundance is consistently observed in metabolic disorders and is associated with a pro-inflammatory state and compromised gut barrier function [50]. Mechanistically, Proteobacteria can contribute to metabolic diseases through several pathways [50,96,133]. The production of lipopolysaccharides (LPSs) triggers systemic inflammation by activating Toll-like receptor-4 (TLR-4) in mice with increased endotoxemia, promoting insulin resistance through the release of cytokines such as interleukin-1 (IL-1), interleukin-6 (IL-6), and tumor necrosis factor-alpha (TNF-α) [134]. LPSs also affect the gut–brain axis by activating microglia involved in satiety regulation in vivo [135]. Additional mechanisms linking Proteobacteria to metabolic diseases include the disruption of gut barrier integrity, facilitating the translocation of microbial products and toxins, and the generation of virulence factors and metabolites, such as colibactin, outer-membrane vesicles, and uremic toxins, which further promote inflammation and metabolic dysfunction [50,96,133]. Furthermore, the production of trimethylamine N-oxide (TMAO), a metabolite strongly associated with increased metabolic and cardiovascular disease, promotes metabolic dysfunction by inducing endothelial dysfunction and endoplasmic reticulum stress, impairing β-cell function and glucose-stimulated insulin secretion, and activating inflammasome pathways in adipose tissue, leading to inflammation, fibrosis, and insulin resistance [136,137].

MASLD is a prominent and clinically significant condition within the realm of metabolic disorders. Patients with MASLD exhibit distinct alterations in gut microbiome composition, characterized by an increased prevalence of Proteobacteria, including genera such as *Escherichia*, *Serratia*, and *Aggregatibacter*, together with a reduction in beneficial taxa belonging to different phyla, such as *Akkermansia* and *Faecalibacterium* [138,139,140]. Proteobacteria overgrowth may promote the development of MASLD through two distinct mechanisms: first, by increasing the risk of insulin resistance, a key pathway in the pathogenesis of this disorder; second, by promoting direct liver damage driven by LPS production [138]. Furthermore, the production of LPS may represent a key determinant of the onset and progression of MASLD, by promoting direct liver damage as well as inducing systemic inflammation and insulin resistance. This process activates hepatic TLR-4 and NF-κB signaling, thereby promoting chronic inflammation, hepatocyte injury, and fibrogenesis [141,142]. Additionally, metabolites derived from Proteobacteria, such as ethanol and secondary bile acids, further disrupt hepatic metabolic homeostasis and promote steatosis and fibrotic remodeling [143].

ABS, also known as gut fermentation syndrome, represents a distinct clinical condition characterized by the endogenous production of ethanol, frequently by *K. pneumoniae* or *Candida* spp., causing symptoms of alcohol intoxication [10,144,145]. In ABS, the endogenous ethanol production results in symptoms that mimic alcohol intoxication [146] (Figure 2). Treatment typically includes dietary adjustments and antimicrobial therapy. However, antibiotic-resistant strains of *K. pneumoniae* and *E. coli* emphasize the need for alternative therapies [147]. Recurrent urinary tract infections (rUTIs), mostly caused by *E. coli* and *K. pneumoniae*, also exemplify the impact of Proteobacteria dysbiosis [148]. Patients with gastrointestinal symptoms often exhibit damage to the epithelial barrier, which is associated with a high abundance of Proteobacteria and a reduction in SCFA-producing microbes [149,150]. Restoring balance through diet, prebiotics, and probiotics may help prevent rUTIs and reduce reliance on antibiotics [151]. FMT has shown promise in treating multidrug-resistant (MDR) infections, including rUTIs, highlighting the therapeutic potential of microbiota modulation [152,153].

## 5. Proteobacteria as Diagnostic and Therapeutic Tools

Proteobacteria have been implicated in the pathogenesis of numerous diseases, which has naturally drawn scientific interest toward their potential use as disease biomarkers. Among these conditions, IBDs stand out as those where Proteobacteria have shown the greatest diagnostic and prognostic utility, albeit still mostly preliminary. Although much of the current literature on IBD biomarkers emphasizes non-Proteobacteria species such as *Faecalibacterium prausnitzii* [154], quantifying Proteobacteria may offer complementary value. For instance, reduced *F. prausnitzii* abundance can help distinguish IBD from irritable bowel syndrome (IBS), while the combined analysis of *F. prausnitzii* and *E. coli* abundance can differentiate colonic CD from extensive ulcerative colitis (UC) [155]. Following this approach, Zheng et al. recently developed a microbiome-based diagnostic tool for IBDs that encompasses different taxa, including one Proteobacterium, namely *E. coli* [156]. Specific Proteobacteria families also appear to correlate with distinct CD phenotypes. High levels of Aeromonadaceae were associated with penetrating CD, Enterobacteriaceae and Pseudomonadaceae with stricturing CD, and Pseudomonadaceae alongside Enterococcaceae with fistulizing forms [157].

Beyond diagnostics, Proteobacteria may also have prognostic significance. For example, enteropathogenic *E. coli* (EPEC) is correlated with UC severity [158], and the overall Proteobacteria phylum is positively associated with disease severity in both UC and CD [157]. Additionally, increased levels of Proteobacteria in the ileal mucosa—alongside decreased Firmicutes—could predict post-operative endoscopic recurrence in CD patients [159]. In CRC, however, the biomarker potential of Proteobacteria remains unclear. CRC-associated microbiota often exhibits an increase in Proteobacteria and *F. nucleatum*, with a concurrent reduction in SCFA-producing bacteria [160]. Since no single Proteobacteria species has emerged as a reliable diagnostic marker, and because *F. nucleatum* and certain Firmicutes are more consistently implicated, these latter taxa currently dominate the field as diagnostic biomarkers for CRC [122,161].

Nevertheless, Proteobacteria are increasingly being investigated for their potential prognostic value in CRC. In a cohort of over 500 Chinese CRC patients, Xu et al. stratified individuals into two microbiota-based clusters. The group enriched with Proteobacteria exhibited significantly worse survival outcomes [162]. Furthermore, intratumoral Proteobacteria may provide prognostic insights; for example, colibactin-producing *E. coli*, which is typically found in more aggressive CRC forms [163], fosters a tumor microenvironment with reduced immunogenicity, facilitating immune evasion and resistance to immunotherapy, particularly in stage III–IV disease [164]. Similarly, *K. pneumoniae* strains expressing a specific isoform of cytidine deaminase can inactivate gemcitabine—a chemotherapeutic agent used in CRC and pancreatic cancer—conferring drug resistance to the patient. This resistance has been shown to be reversible with ciprofloxacin treatment [165]. Building on these findings, the ongoing French clinical trial “MICARE” aims to identify fecal microbial signatures predictive of response to neoadjuvant therapy in rectal cancer, including genotoxin-producing strains such as colibactin-producing *E. coli* [166].

Despite their association with disease, Proteobacteria may also show therapeutic potential. For instance, the probiotic strain *E. coli* Nissle 1917 has been shown to competitively inhibit pathogenic *E. coli* through nutrient competition, supporting its use in UC management [167]. Moreover, *E. coli* has become one of the most frequently engineered bacterial species in microbiome research. Recently, Zou et al. genetically modified *E. coli* Nissle 1917 to act as a biosensor of IBD activity: the engineered strain was able to detect colonic thiosulfate (a marker of inflammation) in mice and produce a detectable colorimetric signal in feces [168].

Another example of microbial engineering involves *Acinetobacter baylyi*, which was engineered to recognize colon cancer cells by activating a kanamycin-resistance gene upon contact. This mutation can be detected through a simple antibiogram of fecal samples [169].

Although historically considered primarily pathogenic, Proteobacteria may also hold promise as diagnostic and therapeutic tools, opening new avenues for disease detection, monitoring, and intervention.

## 6. Therapeutic Strategies Against Proteobacteria

Over recent decades, a growing array of microbiota-targeted therapeutic strategies has been developed to re-establish microbial homeostasis and attenuate inflammation [13]. These approaches range from conventional modalities—such as dietary interventions, antibiotics, prebiotics, probiotics, and postbiotics—to more innovative strategies, including fecal microbiota transplantation (FMT), bacteriophage-based treatments, and engineered microbial therapeutics. Collectively, despite substantial heterogeneity in the available evidence, these interventions are advancing the potential for personalized manipulation of the gut microbiome, offering promising therapeutic avenues for both communicable and non-communicable diseases.

### 6.1. Diet

Dietary patterns represent one of the most powerful modulators of the gut microbiome. The Western diet—characterized by high intake of saturated fats and refined sugars and low fiber content—is strongly associated with dysbiosis, expansion of Proteobacteria (particularly Enterobacteriaceae), and reduced microbial diversity. Conversely, dietary profiles rich in fiber, polyphenols, and unsaturated fatty acids, such as the Mediterranean diet, promote greater microbial diversity, increase the abundance of beneficial taxa (e.g., *Akkermansia*, *Faecalibacterium*), and suppress Proteobacteria proliferation [170,171].

### 6.2. Antibiotics

Over the years, different antimicrobial agents have been introduced in clinical practice to counteract the overexpansion of Proteobacteria and the resulting damage. Among these, beta-lactams (including penicillins, cephalosporins, and carbapenems), fluoroquinolones, aminoglycosides, macrolides, and cotrimoxazole are the most commonly used [172]. By targeting key processes in Proteobacteria metabolism—such as genomic replication, ribosomal activity, and cell wall synthesis [173]—these medications have demonstrated both bacteriostatic and bactericidal effects across a broad range of clinical settings, like meningococcal meningitis, pertussis, gonorrhea, and Enterobacteriaceae gastroenteric infections [174,175,176]. As a consequence, the extensive use of these molecules over the years has led to a sharp increase in multidrug-resistant bacteria (MDR), thereby diminishing the therapeutic efficacy of antibiotics [177].

The Global Antimicrobial Resistance and Use Surveillance System (GLASS) 2022 report [178] highlighted the widespread distribution of antimicrobial resistance worldwide. Based on data reported by 87 countries in 2020, high levels of resistance were observed among different taxa belonging to Proteobacteria, including *E. coli*, *K. pneumoniae*, and *Acinetobacter* spp. Within this framework, resistance to commonly used antibiotics was particularly concerning among Enterobacteriaceae, with resistance of *E. coli* to third-generation cephalosporins and fluoroquinolones exceeding 50% in many high-income countries. Likewise, resistance of *K. pneumoniae* to carbapenems exceeds 50% in many countries. These observations support translational pharmaceutical research aimed at identifying and developing new targeted compounds that could overcome the above-mentioned resistance mechanisms, such as darobactins [179]. In addition, although systemic antibiotic therapy is well established in treating infectious diseases, it is also known to cause significant disruption of the gut microbiome composition, by reducing microbial diversity, depleting beneficial taxa, and potentially disrupting metabolic and ecological networks that provide colonization resistance against pathobionts [180]. Such perturbations may select ecological niches that likely foster an enrichment in pathogenetic Proteobacteria, perpetuating the dysbiotic state that antimicrobial treatment was initially intended to control [180]. However, non-absorbable agents such as rifaximin have emerged in clinical practice as valuable tools for targeted microbiome modulation. Rifaximin exerts a relatively selective and transient effect on Proteobacteria—especially Enterobacteriaceae and other potential enteropathogens—while largely preserving overall microbial diversity in most clinical contexts [181,182]. Its influence on Proteobacteria is generally modest and reversible, with microbial abundances typically returning to baseline after discontinuation [182]. Beyond its direct antimicrobial activity, rifaximin also modulates bacterial virulence, reduces microbial translocation, and shapes host immune responses, including downregulation of pro-inflammatory cytokines and preservation of epithelial barrier integrity [181].

### 6.3. Prebiotics, Probiotics, and Postbiotics

Prebiotics, probiotics, and postbiotics contribute to the establishment of a eubiotic microbial ecosystem by limiting the overgrowth of Proteobacteria overgrowth and reducing the risk of metabolic and inflammatory disorders through the restoration of gut homeostasis and the attenuation of pro-inflammatory microbial metabolites [183]. Prebiotics are nondigestible dietary substrates that selectively promote the growth and metabolic activity of specific gut microorganisms [184]. By preferentially promoting beneficial taxa such as *Bifidobacterium* and *Lactobacillus*, prebiotics reduce the abundance of opportunistic and conditionally pathogenic Proteobacteria through competitive exclusion. This shift is accompanied by increased SCFA production, acidification of the colonic environment, and enhanced integrity of the epithelial barrier. Together, these changes inhibit the proliferation of pathogenic Proteobacteria and reduce endotoxemia [185,186]. Probiotics are defined as live microorganisms that, when administered in adequate quantities, confer health benefits to the host [187]. Strains belonging to *Lactobacillus* and *Bifidobacterium* are particularly effective at suppressing Proteobacteria through bacteriocin production, competitive exclusion, and modulation of mucosal immune responses [188,189]. Postbiotics, which consist of inanimate microorganisms and/or their cellular components, also provide clinically relevant health benefits [183]. They exhibit anti-inflammatory and antimicrobial properties, strengthen epithelial barrier function, and directly inhibit Proteobacteria through bioactive metabolites such as SCFAs, bacteriocins, and exopolysaccharides [183,190]. However, despite an increasingly clear understanding of their pharmacodynamic mechanisms, the use of prebiotics, probiotics, and postbiotics remains largely empirical to date. Nevertheless, the increasingly widespread implementation of microbiome testing in clinical practice, enabling a metagenome-informed therapeutic manipulation of gut microbiota, could facilitate their use for the targeted modulation of Proteobacteria.

### 6.4. Fecal Microbiota Transplantation

Fecal microbiota transplantation (FMT) involves the administration of processed stool from a healthy donor to a recipient in order to restore a disrupted gut microbial community [191]. Currently, FMT is formally approved for clinical use exclusively in the treatment of recurrent *Clostridioides difficile* infection [192]. Nevertheless, multiple studies have shown that in disorders characterized by dysbiosis, FMT enhances microbial diversity and shifts the gut community toward a composition resembling that of healthy donors, accompanied by a substantial reduction in Proteobacteria—particularly Enterobacteriaceae and other pathobionts [193,194]. These effects are driven by the direct engraftment of donor taxa, competitive displacement of Proteobacteria, and the reconstitution of functional microbial networks, including those involved in SCFA synthesis and bile acid metabolism [195]. Moreover, a growing body of evidence indicates that FMT may facilitate the decolonization of carriers of multidrug-resistant Enterobacteriaceae, with encouraging outcomes [196,197,198]. Hence, further clinical studies are warranted to investigate whether FMT might represent a valuable option in the management of disorders characterized by an enrichment in Proteobacteria.

### 6.5. Artificial Microbiome Therapeutics: Bacteriophage Therapy and Engineered Bacteria

The use of the aforementioned therapeutic microbiome modulators is limited by several factors such as poor precision, regulatory challenges, and the inability to deliver consistent treatments. To overcome these obstacles, artificial microbiome therapeutics have been developed, including microbiota consortia, bacteriophages, and engineered probiotics. These are designed to be consistent, targeted, and safe for modulating the human gut microbiota [13].

Microbiota consortia are advanced products derived from FMT, defined as multi-strain communities of commensal bacteria [199,200]. Recent evidence indicates that tailored consortia can decolonize Proteobacteria and reduce inflammation in preclinical models through competitive exclusion and metabolic regulation [201,202].

Bacteriophages, viruses that specifically target bacteria, offer precise modulation of the microbiome [193]. Preclinical studies have demonstrated that bacteriophages can selectively deplete pathogenic Proteobacteria, such as *K. pneumoniae* and *Enterobacter cloacae*, while maintaining the overall structure of the microbiota [203,204,205].

Engineered probiotics are genetically modified strains designed to perform targeted functions, such as producing antimicrobial peptides and modulating the immune system [191,201]. Pre-clinical studies show they can reduce the abundance of Proteobacteria and enhance mucosal barrier function while preserving commensal populations [206,207]. At present, most evidence regarding artificial microbiome therapeutics comes from preclinical research, and their integration into clinical practice remains distant. Nonetheless, this approach represents a therapeutic modality with considerable promise for future clinical applications.

## 7. Conclusions

Proteobacteria represent a significant component of the human microbiota, playing roles that range from early-life colonization and nutrient cycling to inflammation, infection, and cancer. Their metabolic versatility and ability to thrive in oxygen-rich or inflamed environments provide them with a competitive edge during dysbiosis. However, this same adaptability contributes to the involvement of specific taxa of Proteobacteria in various chronic diseases, including inflammatory bowel disease, colorectal and gastric cancers, and metabolic conditions such as MASLD and obesity.

Key species within this phylum, such as adherent-invasive *E. coli* and *H. pylori*, exemplify how Proteobacteria can both exploit and exacerbate host inflammation, promoting mucosal barrier disruption, immune activation, and even DNA damage. Their interactions with the host are multifaceted: they benefit from inflammation-induced shifts in oxygen availability and nutrient composition, while simultaneously driving pathological changes through genotoxins like colibactin and inflammatory mediators such as LPS.

The clinical implications of Proteobacteria extend beyond their role in pathogenesis. Increasing evidence may support their use in the future as biomarkers for disease diagnosis, prognosis, and therapeutic monitoring, particularly in IBD and CRC. Moreover, some initial pivotal studies are seeing Proteobacteria emerge as promising tools in precision medicine: engineered strains, bacteriophage therapies, and targeted dietary interventions are being developed to harness or counteract their influence on the host.

Ultimately, the dual nature of Proteobacteria, as both pathogens and potential allies, highlights the need for nuanced therapeutic approaches. Modulating their abundance, virulence, or ecological niche, rather than attempting wholesale removal, may provide a more effective strategy for restoring microbial balance and reducing the impact of disease. Continued research into their metabolic networks, host interactions, and role in dysbiosis will be critical for translating microbiome science into effective clinical interventions. Once viewed with caution, Proteobacteria may thus become central players in the future of microbiota-based medicine.

## Figures and Tables

**Figure 1:**
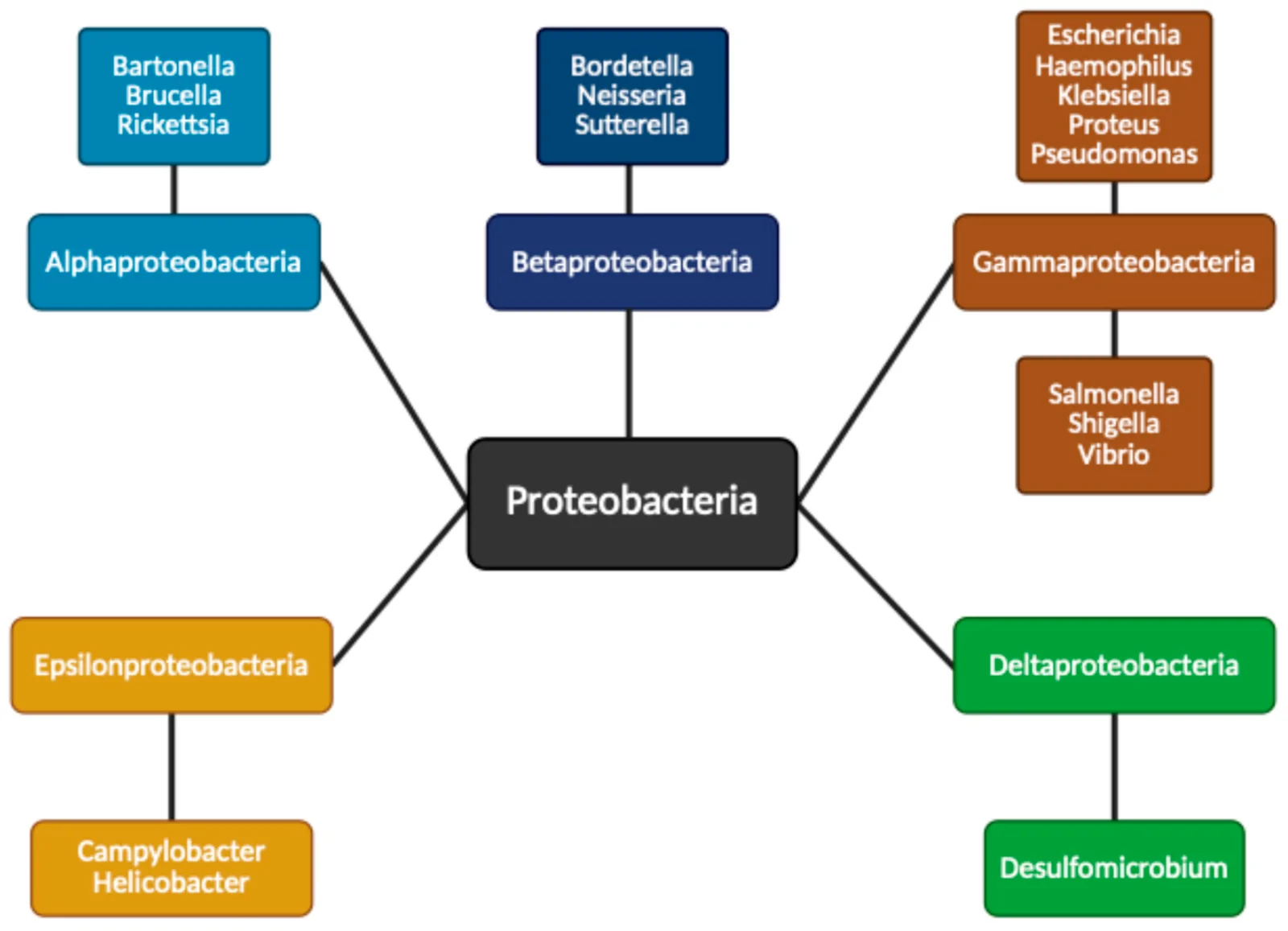
Taxonomy of clinically relevant Proteobacteria: this figures includes the classes and genera of Proteobacteria (currently also known as “Pseudomonadota”) more frequently faced in clinical practice.

**Figure 2:**
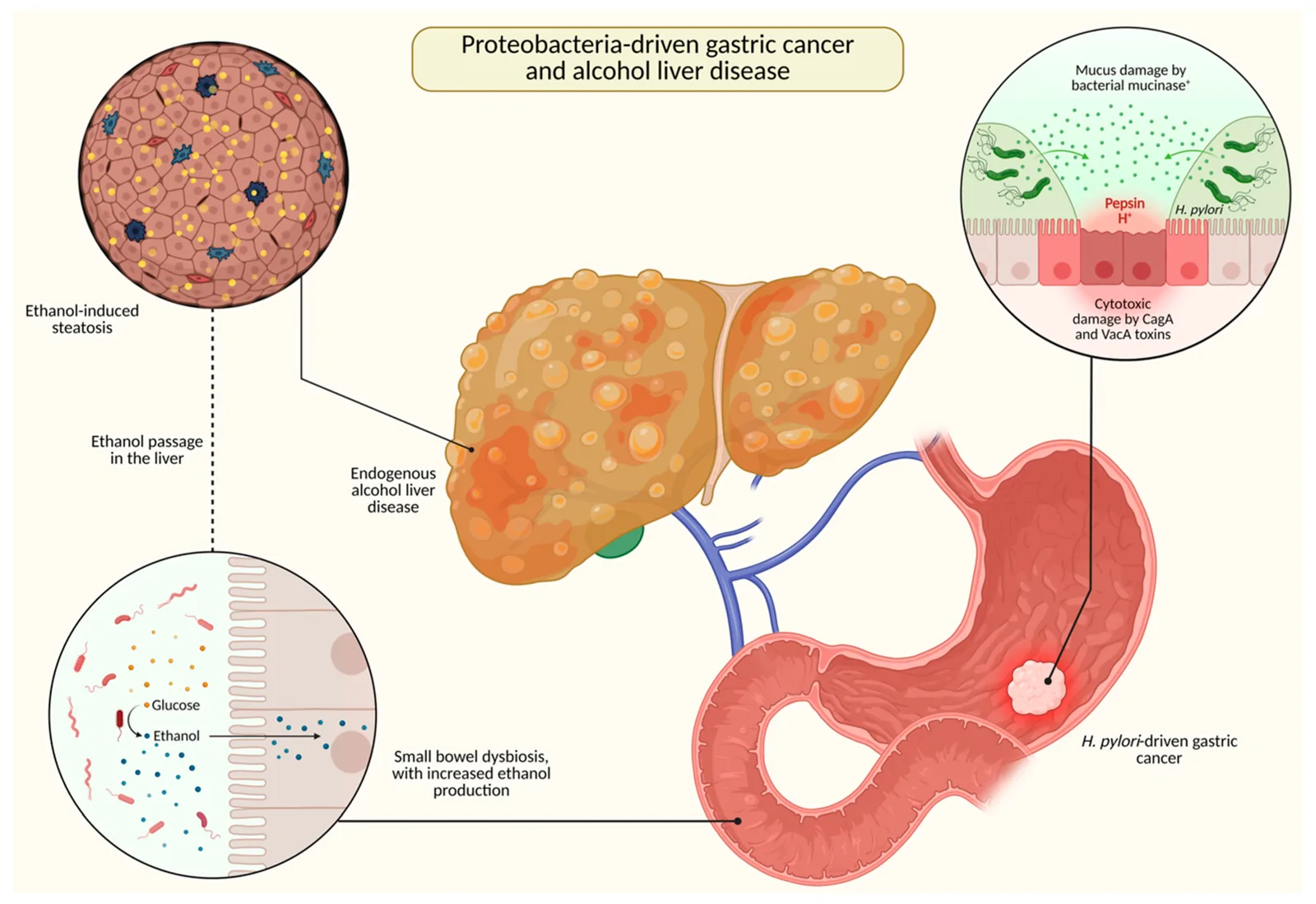
Proteobacteria-driven gastric cancer and alcoholic liver disease. Many diseases see Proteobacteria as key etiopathogenetic agents: gastric cancer and bacterial autobrewery syndrome are paradigmatic. Gastric cancer can be typically due to *H. pylori* infection. While its survival in the gastric mucus is guaranteed by its mucinases, CagA and VacA cytotoxins are able to damage the gastric mucosa, leading, eventually, to the development of gastric cancer. Autobrewery syndrome is caused by an increase in the endogenous ethanol production. This may happen in the case of an excessive abundance of ethanol-producing bacteria, like *K. pneumoniae*, in the small intestine. This ethanol, being absorbed by enterocytes, then reaches the portal system and then the liver. Here it enhances a condition of fatty liver, similar to the more known exogenous alcohol liver disease, damaging structures and functions of the hepatocytes. *H. pylori*, *Helicobacter pylori*; CagA, cytotoxin-associated gene A; and VacA, vacuolating cytotoxin A.

**Table 1:** Principal Proteobacteria species pathogenetically linked with disease.

Species	Related Diseases		Ref.
*Sutterella wadsworthensis*	Obesity	Presence of *S. wadsworthensis* in the gut microbiota has been positively correlated with obesity in children and adolescents	[62]
	Autism	*S. wadsworthensis* has been found in intestinal biopsy samples from children with autism and gastrointestinal symptoms	[63]
*Escherichia coli*	Inflammatory bowel disease	*E. coli* has been highlighted at the mucosal level in inflammatory bowel disease patients, especially in those with ulcerative colitis	[64]
	Colorectal cancer	Colibactin-producing *E. coli* seemingly enhances tumor growth, as shown in in vivo and in vitro models	[65]
	Obesity and type 2 diabetes	*E. coli* likely aggravates impaired glucose homeostasis and energy metabolism in mice	[66]
	Atherosclerosis	An enrichment of *E. coli* in the gut microbiota has been documented in patients with carotid atherosclerosis	[67]
	Parkinson’s disease	Administration of *E. coli* has been linked with α-synuclein accumulation in the colon and spread to the brain via the gut–brain axis in mice	[68]
*Klebsiella pneumoniae*	Inflammatory bowel disease	*K. pneumoniae* might contribute to intestinal inflammation in ulcerative colitis patients through activation of caspase-11 inflammasomes	[69]
	Hepatocellular carcinoma	Enrichment of *K. pneumoniae* is associated with increased cell proliferation and activation of oncogenic signaling in mice	[70]
	Hypertension	*K. pneumoniae* seemingly induces higher blood pressure and slight cardiac hypertrophy in mice	[71]
	Acute myocardial infarction	Abundance of *K. pneumoniae* results are increased in patients with acute myocardial infarction and positively associated with disease status and severity	[72]
	Ankylosing spondylitis	Antibodies against an epitope of *K. pneumoniae* have been found in patients with ankylosing spondylitis	[73]
	Alzheimer’s disease	Translocation of *K. pneumoniae* from the gut has been associated with impaired neurobehavioral function and elevated total tau levels in mice	[74]
*Proteus mirabilis*	Inflammatory bowel disease	*P. mirabilis* results are enriched in the gut microbiota of Crohn’s disease patients, potentially contributing to a more severe colonic inflammation in mice	[75]
	Parkinson’s disease	*P. mirabilis* induces motor deficits, dopaminergic neuronal damage, and α-synuclein aggregation in mice	[76]
*Pseudomonas aeruginosa*	Irritable bowel syndrome	*P. aeruginosa* has been found to be more prevalent and abundant in irritable bowel syndrome patients than in healthy controls	[77]
	Atherosclerosis	Infection with *P. aeruginosa* stimulates atherogenesis in rats fed a cholesterol-supplemented diet	[78]
	Alzheimer’s disease	*P. aeruginosa* pulmonary infection triggers the release of cytotoxic tau from lung endothelial cells, leading to neuronal tauopathy in mouse models	[79]
	Parkinson’s disease	Rhamnolipid, an amphiphilic molecule produced by *P. aeruginosa*, induces α-synuclein fibrillation in vitro	[80]
*Desulfomicrobium orale*	Periodontal disease	*D. orale* has been isolated from periodontal pockets and may contribute to periodontal tissue damage through the production of cytotoxic hydrogen sulfide.	[25]
*Campylobacter jejuni*	Irritable bowel syndrome	*C. jejuni* infection might lead to post-infectious irritable bowel syndrome through increased adhesion, invasion, and pro-inflammatory cytokines production	[81]
	Reactive arthritis	Following *C. jejuni* infection, acute reactive arthritis can be developed especially in adult patients	[82]
	Guillain-Barré syndrome	Lipo-oligosaccharides from *C. jejuni* trigger the production of cross-reactive anti-ganglioside antibodies, potentially contributing to the development of Guillain-Barré syndrome	[83]
*Helicobacter pylori*	Peptic ulcer disease	CagA-positive *H. pylori* is linked to increased risk of peptic ulcer disease	[84]
	Gastric cancer	*H. pylori* infection increases risk of gastric cancer, especially non-cardia gastric cancer	[85]
	Colorectal cancer	*H. pylori* seemingly induces pro-carcinogenic STAT3 signaling and loss of goblet cells, contributing to the development of colorectal cancer in mice	[86]
	Insulin resistance	*H. pylori* infection might increase HbA1c levels, therefore having a role in glycemic control	[87]
	Atherosclerosis and obesity	*H. pylori* infection has been linked to increased BMI, triglyceride levels, and risk of carotid plaque formation	[88]
	Hypertension	*H. pylori* infection has been correlated with an increased prevalence of hypertension	[89]
	Rheumatoid arthritis	*H. pylori* infection seemingly induces protein citrullination, potentially contributing to rheumatoid arthritis progression	[90]
	Alzheimer’s disease	*H. pylori* has been shown to stimulate Alzheimer’s disease-associated risk gene expression and trigger neuroinflammation in in vitro models	[91]

## Data Availability

No new data were created or analyzed in this study. Data sharing is not applicable to this article.
